# Supplementary material for: Value of SiPM PET in myocardial perfusion imaging using Rubidium-82
Source: J Nucl Cardiol. 2020 May 14;29(1):204–12. doi: 10.1007/s12350-020-02141-0 (PMC8873116; doi:10.1007/s12350-020-02141-0)
Supplement: Supplementary file 1 — Electronic supplementary material 1 (PPTX 2593 kb) [file 12350_2020_2141_MOESM1_ESM.pptx]

## Slide 1
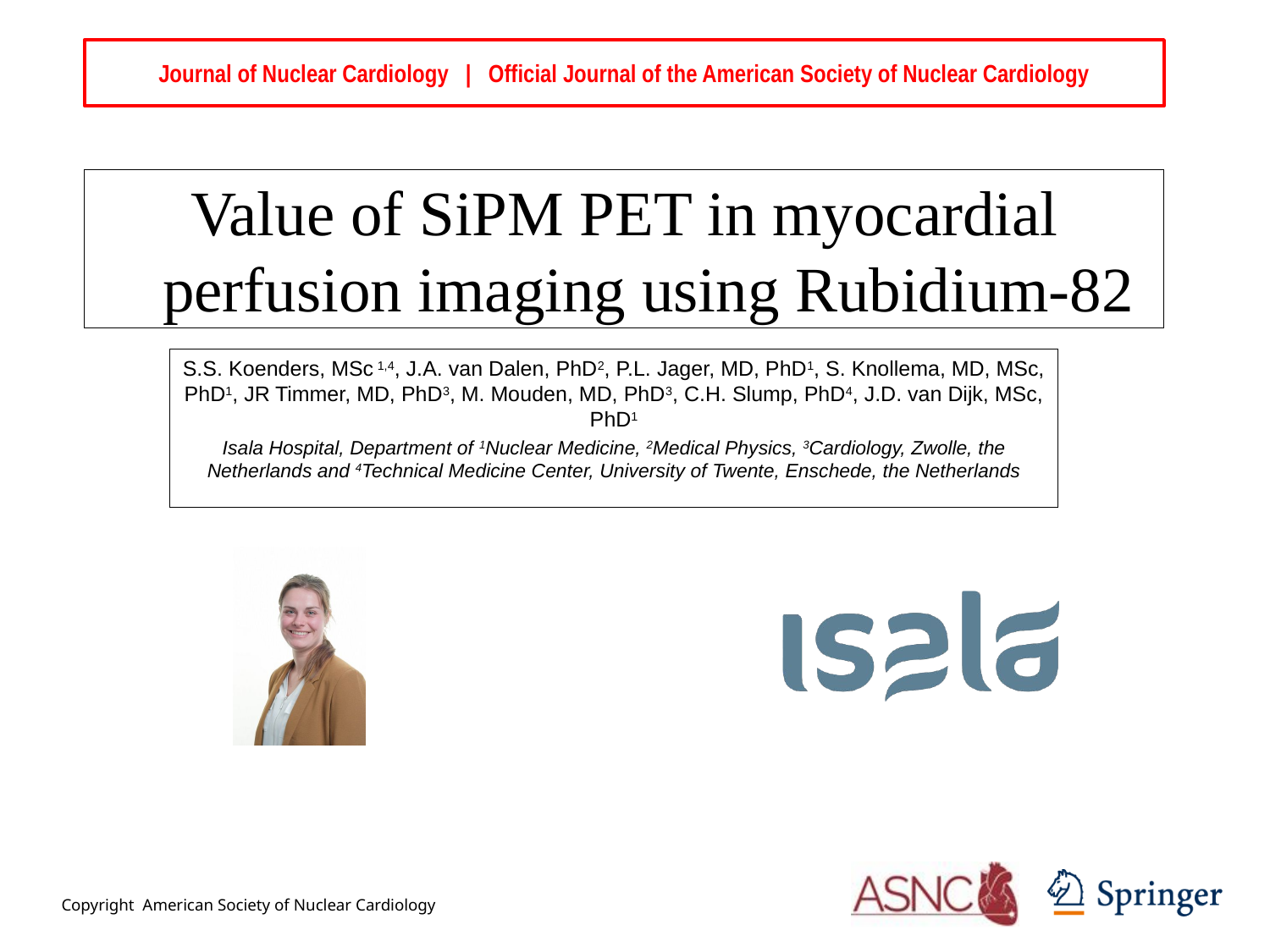

Journal of Nuclear Cardiology | Official Journal of the American Society of Nuclear Cardiology
# Value of SiPM PET in myocardial perfusion imaging using Rubidium-82
S.S. Koenders, MSc 1,4, J.A. van Dalen, PhD2, P.L. Jager, MD, PhD1, S. Knollema, MD, MSc, PhD1, JR Timmer, MD, PhD3, M. Mouden, MD, PhD3, C.H. Slump, PhD4, J.D. van Dijk, MSc, PhD1
Isala Hospital, Department of 1Nuclear Medicine, 2Medical Physics, 3Cardiology, Zwolle, the Netherlands and 4Technical Medicine Center, University of Twente, Enschede, the Netherlands
Copyright American Society of Nuclear Cardiology

## Slide 2
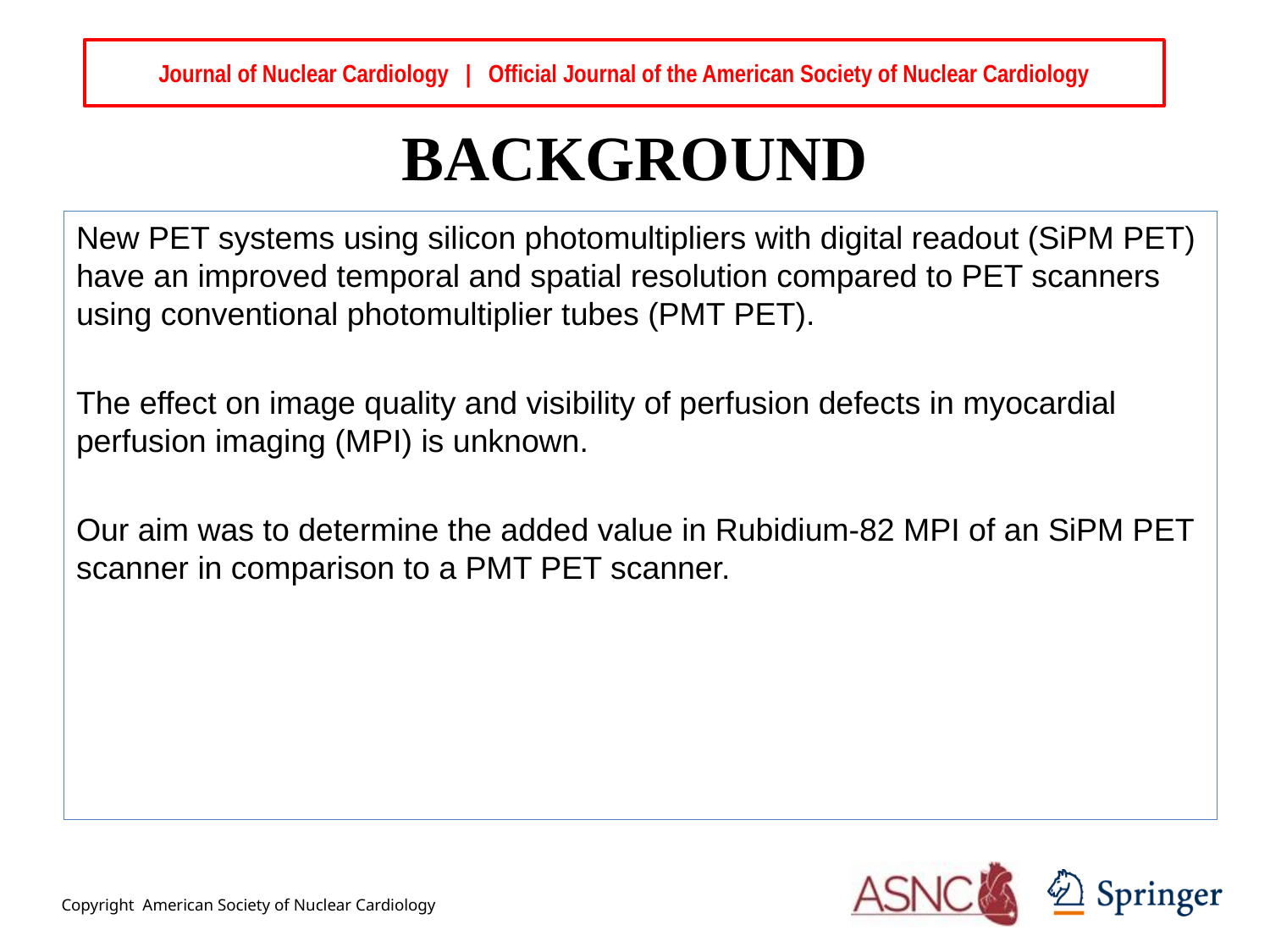

Journal of Nuclear Cardiology | Official Journal of the American Society of Nuclear Cardiology
# BACKGROUND
New PET systems using silicon photomultipliers with digital readout (SiPM PET) have an improved temporal and spatial resolution compared to PET scanners using conventional photomultiplier tubes (PMT PET).
The effect on image quality and visibility of perfusion defects in myocardial perfusion imaging (MPI) is unknown.
Our aim was to determine the added value in Rubidium-82 MPI of an SiPM PET scanner in comparison to a PMT PET scanner.
Copyright American Society of Nuclear Cardiology

## Slide 3
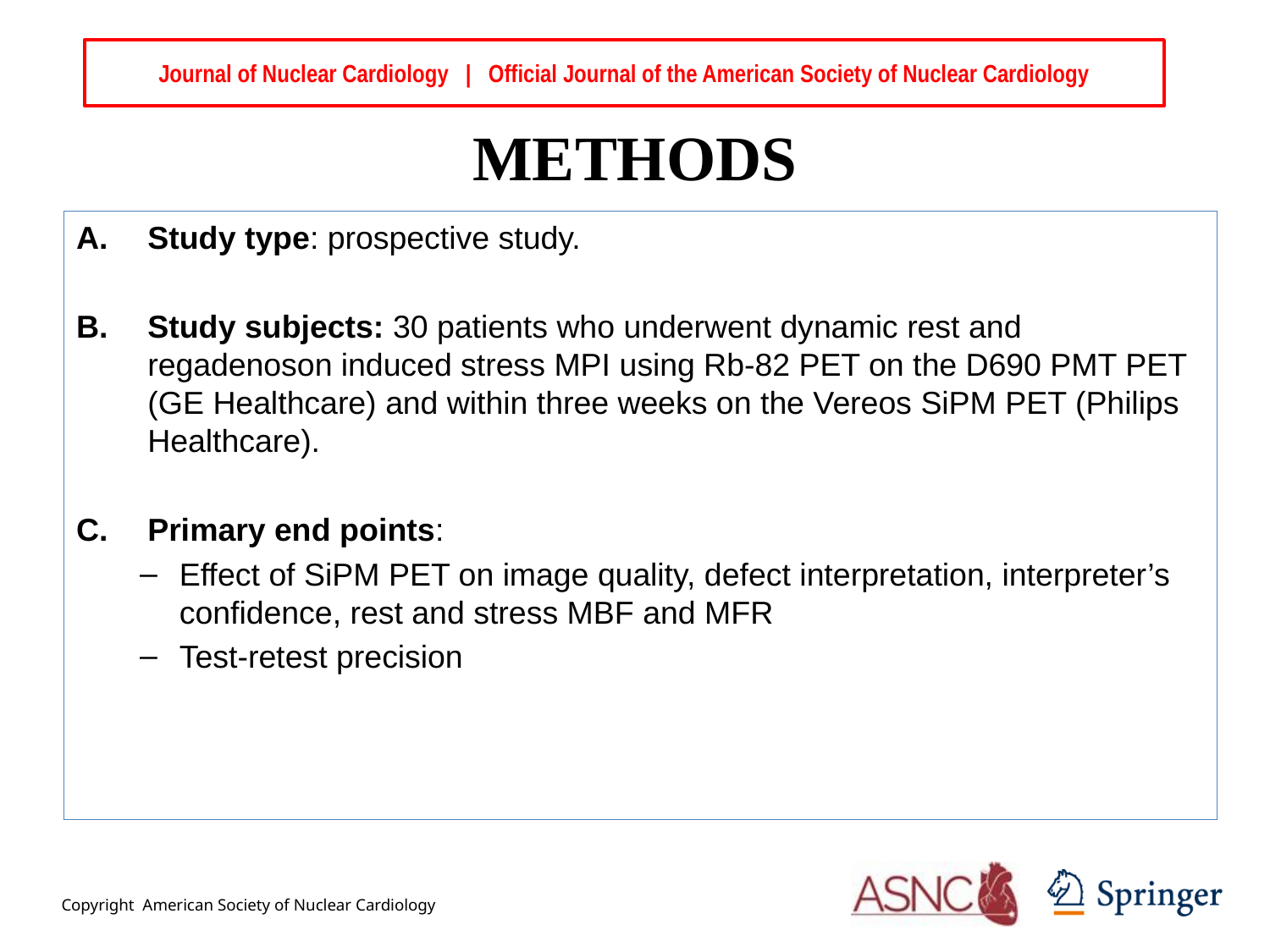

Journal of Nuclear Cardiology | Official Journal of the American Society of Nuclear Cardiology
# METHODS
Study type: prospective study.
Study subjects: 30 patients who underwent dynamic rest and regadenoson induced stress MPI using Rb-82 PET on the D690 PMT PET (GE Healthcare) and within three weeks on the Vereos SiPM PET (Philips Healthcare).
Primary end points:
Effect of SiPM PET on image quality, defect interpretation, interpreter’s confidence, rest and stress MBF and MFR
Test-retest precision
Copyright American Society of Nuclear Cardiology

## Slide 4
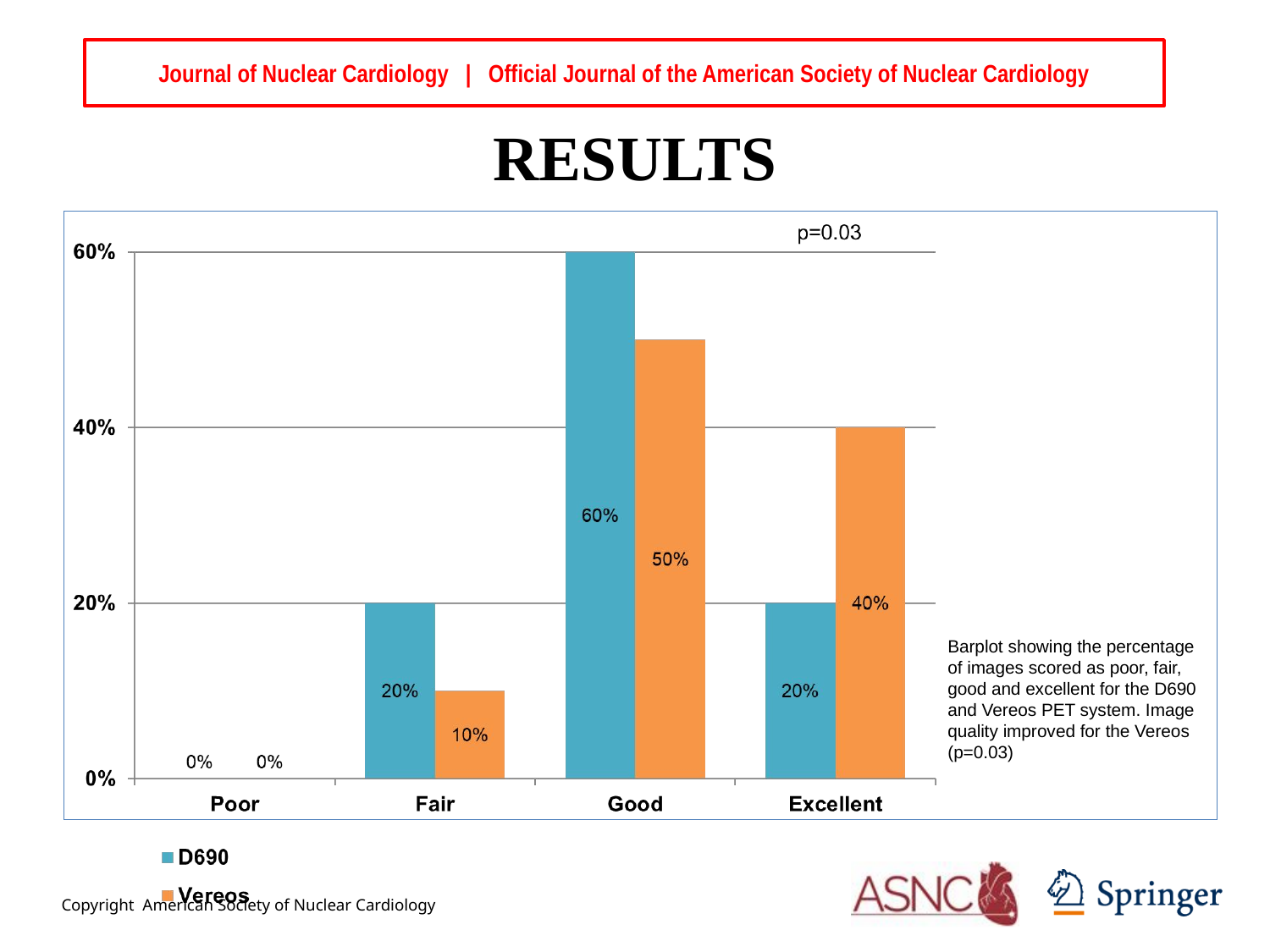

Journal of Nuclear Cardiology | Official Journal of the American Society of Nuclear Cardiology
# RESULTS
Barplot showing the percentage of images scored as poor, fair, good and excellent for the D690 and Vereos PET system. Image quality improved for the Vereos (p=0.03)
Copyright American Society of Nuclear Cardiology

## Slide 5
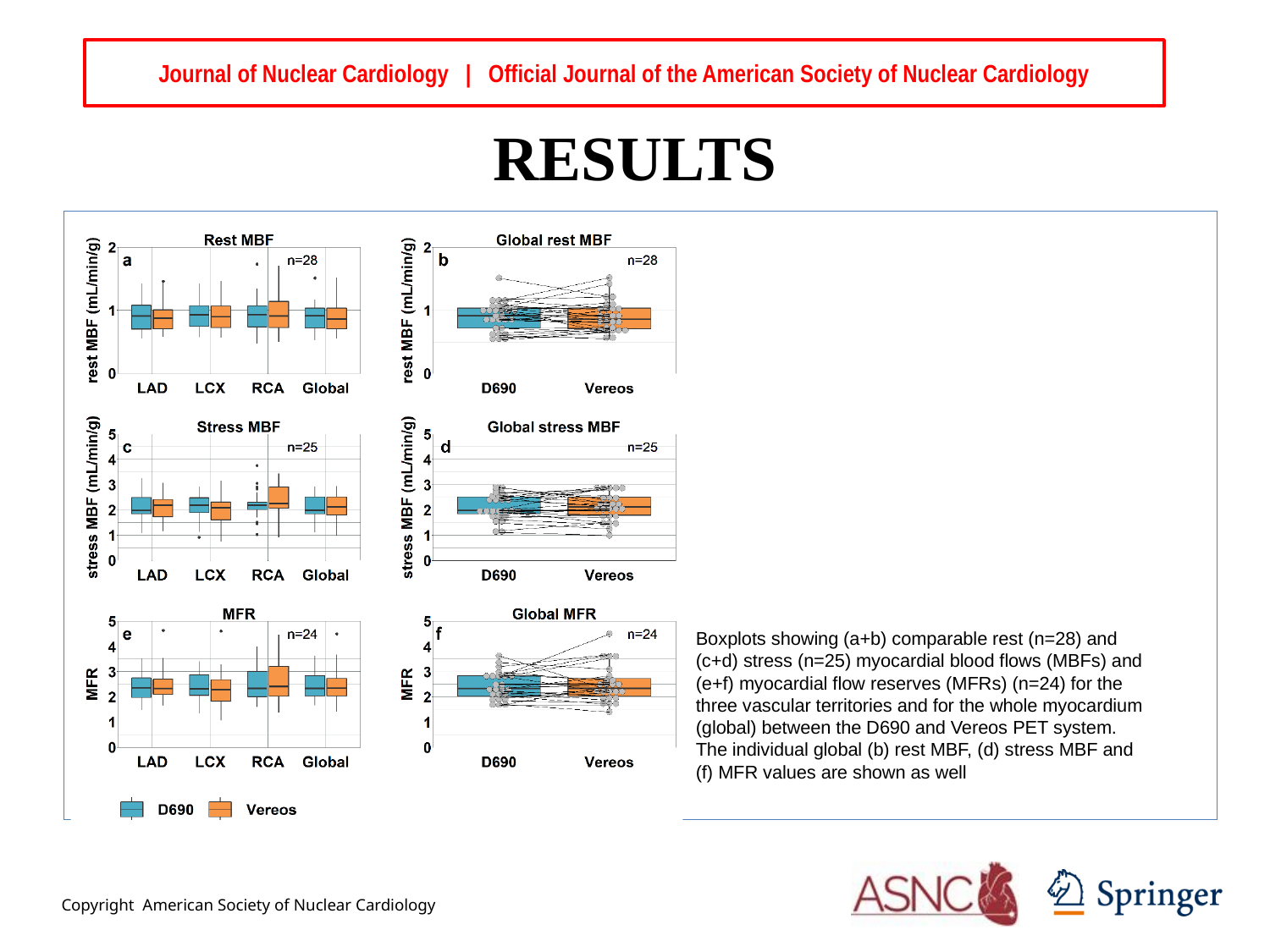

Journal of Nuclear Cardiology | Official Journal of the American Society of Nuclear Cardiology
# RESULTS
Boxplots showing (a+b) comparable rest (n=28) and (c+d) stress (n=25) myocardial blood flows (MBFs) and (e+f) myocardial flow reserves (MFRs) (n=24) for the three vascular territories and for the whole myocardium (global) between the D690 and Vereos PET system. The individual global (b) rest MBF, (d) stress MBF and (f) MFR values are shown as well
Copyright American Society of Nuclear Cardiology

## Slide 6
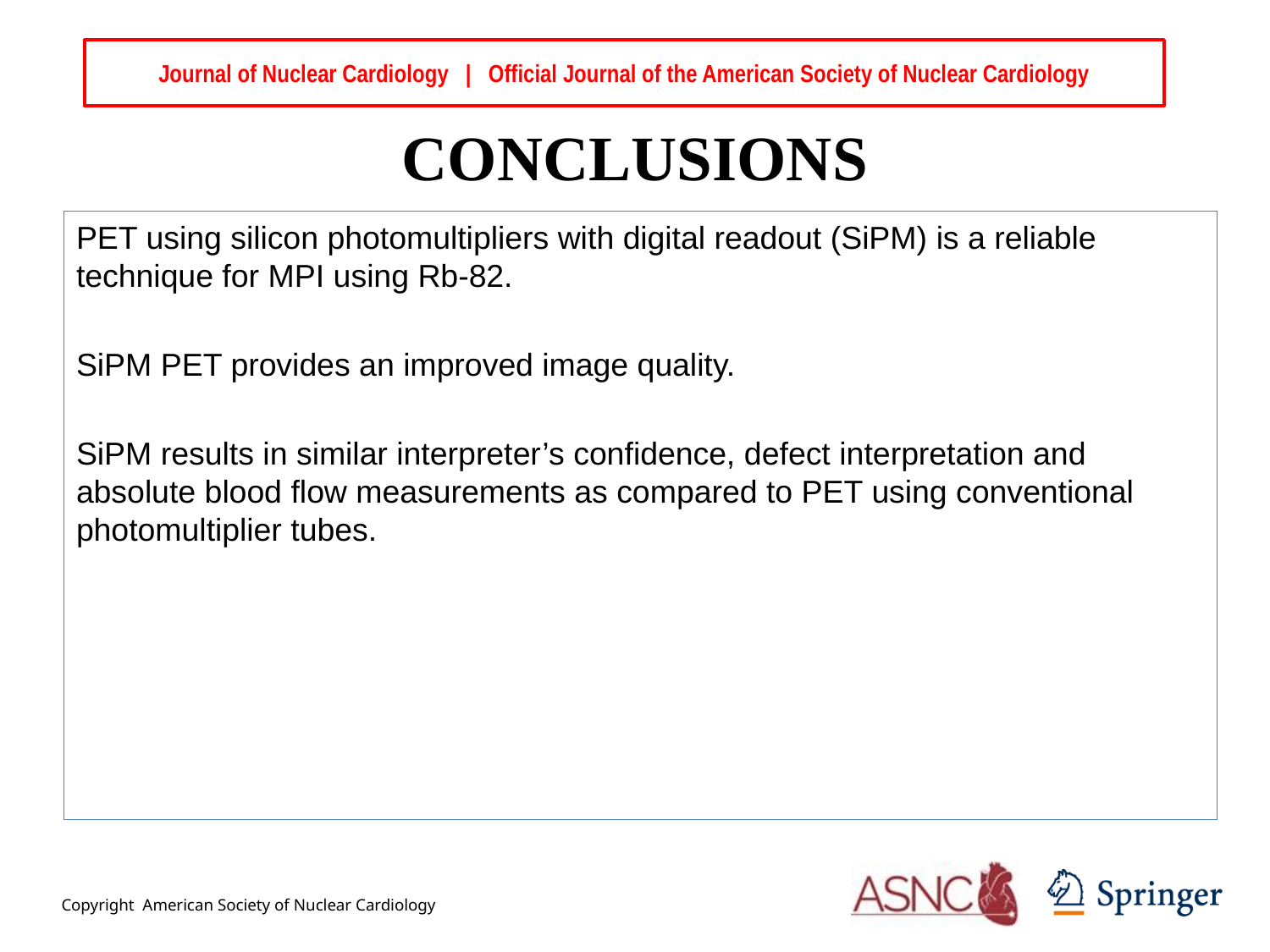

Journal of Nuclear Cardiology | Official Journal of the American Society of Nuclear Cardiology
# CONCLUSIONS
PET using silicon photomultipliers with digital readout (SiPM) is a reliable technique for MPI using Rb-82.
SiPM PET provides an improved image quality.
SiPM results in similar interpreter’s confidence, defect interpretation and absolute blood flow measurements as compared to PET using conventional photomultiplier tubes.
Copyright American Society of Nuclear Cardiology
